# Assessment of Circulating LncRNAs Under Physiologic and Pathologic Conditions in Humans Reveals Potential Limitations as Biomarkers

**DOI:** 10.1038/srep36596

**Published:** 2016-11-18

**Authors:** Kenny Schlosser, Jennifer Hanson, Patrick J. Villeneuve, Jim Dimitroulakos, Lauralyn McIntyre, Louise Pilote, Duncan J. Stewart

**Affiliations:** 1 Regenerative Medicine Program, Ottawa Hospital Research Institute, Ottawa, ON, Canada; 2 Cancer Therapeutics Program, Ottawa Hospital Research Institute, Ottawa, ON, Canada; 3 Department of Thoracic Surgery, The Ottawa Hospital, Ottawa, ON, Canada; 4 Clinical Epidemiology Program, Ottawa Hospital Research Institute, Ottawa, Ontario, Canada; 5 Division of General Internal Medicine, McGill University Health Centre, Montreal, Quebec, Canada; 6 Research Institute, McGill University Health Center, Montreal, Quebec, Canada; 7 Department of Cellular and Molecular Medicine, University of Ottawa, Ottawa, ON, Canada

## Abstract

Long non-coding RNAs (lncRNA) are a new class of regulatory molecules with diverse cellular functions. Recent reports have suggested that extracellular lncRNAs are detectable in human plasma and may serve as biomarkers. Here, we sought to investigate circulating lncRNAs as potential biomarkers for pulmonary arterial hypertension (PAH). Eighty-four lncRNAs, representing some of the most abundant and functionally relevant candidates identified in cellular studies, were assessed via RT-qPCR in plasma from PAH and healthy subjects. However, despite preamplification, the majority of lncRNAs were surprisingly undetectable or sporadically detectable, and showed no differential changes. Systematic characterization of plasma/RNA quality and technical performance via internal and external controls revealed no evidence of RNA degradation or RT-qPCR inhibition, and most lncRNAs were robustly detectable in pulmonary tissue. In plasma, lncRNA levels were the lowest among several different RNA species examined, and this was generalizable to other chronic and acute vascular conditions including coronary artery disease, acute coronary syndrome, and septic shock. In addition, two of three previously reported circulating lncRNA biomarker candidates were not detectable in any of the plasma samples. This study reveals new insight on the relative levels of lncRNAs in circulation, which has important implications for their potential development as biomarkers.

Large scale transcriptional profiling studies have shown that 70–90% of the human genome is transcribed into RNA, though the majority of this RNA exhibits little or no protein coding potential^1^,^2^,^3^. These studies have contributed to the discovery of new classes of RNA that exhibit extraordinary diversity in size, structure and function. Long non-coding RNAs (lncRNAs) are broadly defined as non-protein coding RNA transcripts greater than 200 nucleotides, which serve to distinguish them from the many types of smaller non-coding RNAs such as miRNAs, siRNAs and snoRNAs. Although lncRNAs comprise the majority of non-coding RNAs in the human transcriptome^4^,^5^, they remain among the least well characterized RNA molecules, and this can be attributed to a combination of factors including low expression levels, poor conservation, and high tissue/cell specificity^5^,^6^. Whether most of these lncRNAs are functional, or simply by-products of transcriptional noise, remains a subject of debate^7^. Nevertheless, a small number of studies have identified lncRNAs that play important and diverse regulatory roles under both physiologic and pathologic conditions^8^, including the negative and positive regulation of gene expression, alternative RNA splicing, regulation of siRNA and miRNA, and the modulation of protein localization and activity^9^,^10^,^11^.

While the majority of studies have focused on the intracellular roles of lncRNAs, there is increasing interest in the potential roles of extracellular circulating lncRNAs. These studies build on the concept of circulating microRNAs, which have shown promise as non-invasive biomarkers across a wide spectrum of diseases and altered physiologic states. The release of cellular RNA species into circulation may reflect disease-specific tissue injury and/or remodelling, or potential intercellular signaling. However, the precise mechanisms through which different RNA species are released into circulation have not been elucidated, making it difficult to predict *a priori* whether certain clinical conditions may lead to perturbations in circulating RNAs. Thus far, alterations in plasma (or serum) lncRNA levels have only been reported in several conditions relating to heart failure^12^, acute kidney injury^13^, coronary artery disease (CAD)^14^ and cancer^15^,^16^. Whether circulating lncRNAs are altered in other pathophysiologic settings requires further investigation.

Pulmonary arterial hypertension (PAH) is a rare and fatal disease characterized by elevated pulmonary artery pressures and a progressive deterioration of the pulmonary vasculature. Although the prognosis for PAH is poor, early diagnosis and management can increase the length and quality of life for patients. However, prompt diagnosis remains a challenge because in early stages symptoms may be lacking or nonspecific, and there are currently no biomarkers to facilitate detection. A number of lncRNAs are known to be enriched in endothelial, smooth muscle, and inflammatory cells, and regulate key cellular processes of potential mechanistic importance to PAH (e.g., apoptosis and proliferation), though their ability to serve as biomarkers has not yet been investigated in PAH patients.

Here, we sought to determine whether a strategic selection of lncRNAs were consistently detectable in circulation, and could serve as non-invasive biomarkers of PAH. We used a hypothesis-driven approach and screened 84 specific lncRNA candidates that are among the most abundant and functionally relevant lncRNAs identified in cells to date^17^. We now report that these lncRNAs are quite difficult to detect in circulation compared to other RNA species, and this observation was generalizable to both physiologic and pathologic conditions including PAH and several other types of chronic and acute vascular conditions. The majority of these lncRNAs were either not detectable or only sporadically detectable in plasma using highly sensitive preamplification qPCR strategies, limiting their utility as non-invasive diagnostic tools. This study reveals the technical challenges associated with circulating lncRNA research, and provides useful insight that can inform future studies.

## Results

### Strategic profiling reveals no changes and very low levels of 84 lncRNAs in plasma of PAH and healthy subjects

In contrast to unbiased global profiling, we conducted a strategic hypothesis-driven screen of 84 specific lncRNAs for several reasons. First, these lncRNAs are among the very few that have been empirically validated to have cellular functions^17^, which is a key distinction from the vast majority of ~40,000 lncRNA transcripts that remain uncharacterized, and could represent products of non-specific transcription that would have no possible link to disease activity. A focused screen would therefore reduce the likelihood of identifying false positive associations with spurious transcripts. This is an important consideration given that the high cost of global microarray profiling experiments often necessitate the use of small sample sizes (e.g., n = 3/group^12^,^13^,^14^), which are statistically underpowered. Second, the selected lncRNAs are known to be expressed in vascular, cardiac and/or inflammatory cells that are relevant to PAH pathobiology. Furthermore, these lncRNAs exhibit diverse functions, including roles in cancer mechanisms, which is an area of increasing interest driven by the notion that PAH and cancer may share common pathologic mechanisms^21^, such as underlying transcriptional reprogramming of cell metabolism, inflammation and proliferation. Third, we examined lncRNAs that are among the most highly expressed lncRNAs in cells, and therefore more likely to be detectable in circulation. Finally, we sought to maximize the sensitivity and linear dynamic range of lncRNA quantification by screening with a PCR array platform (instead of microarray), which would not only help to identify changes in lncRNA levels associated with disease, but also provide information on their relative levels in circulation. This was another key consideration because ideal biomarkers should not only report changes in disease activity, but also be readily measurable and reproducible, and this is a characteristic that has not been clearly addressed for plasma lncRNAs in previous studies. Furthermore, the abundance of non-coding RNA species in cells have been shown to roughly correlate with their level of phylogenetic conservation^18^, which has been suggested to be a good proxy for function^19^,^20^. In parallel, understanding the relative abundance of lncRNAs in circulation may thus provide similar clues about their functional significance.

**Figure 1:**
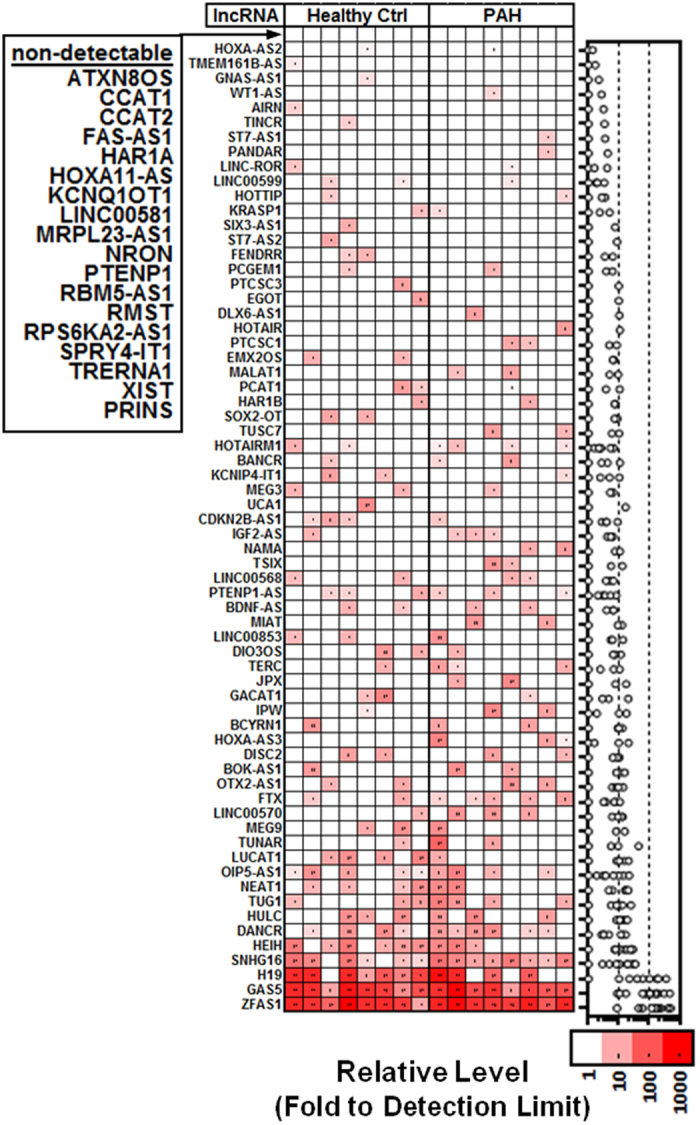
Low and sporadic lncRNA detection in PAH and healthy subject plasma. Heatmap showing color coded relative lncRNA plasma levels expressed fold to the PCR detection limit (Cq = 35; Relative Level = 1) after log transformation. Each column represents a unique subject and each row represents a different lncRNA, which have been arranged arbitrarily in ascending (mean) abundance. LncRNAs that were not detected in any subjects are listed next to the heatmap. Right graph shows relative lncRNA levels of individual subjects (circles).

Plasma lncRNA levels were evaluated in an initial discovery cohort of healthy volunteers and PAH patients closely matched for age and sex (Table 1). Surprisingly, despite a pre-amplification strategy designed to facilitate quantification of low copy number transcripts, only the GAS5 and ZFAS1 lncRNAs were detected in all subjects (between 10–1000 fold above the PCR detection limit (Cq < 35). A total of 64 lncRNAs were detected only sporadically at levels less than 100 fold above the PCR detection limit, and 18 lncRNAs were not detected in any subjects (Fig. 1). No significant differences in plasma lncRNA levels were observed between the PAH and healthy control groups. Very low circulating levels raise important questions as to whether these lncRNAs could serve as practical biomarkers for molecular diagnostic applications. To better interpret these results, we systematically validated the quality and technical performance of our plasma specimens, RNA extraction, reverse transcription and PCR strategy to exclude the possibility that technical artifacts might have contributed to the low lncRNA levels.

### Systematic characterization of specimen quality reveals no evidence of RNA degradation

**Figure 2:**
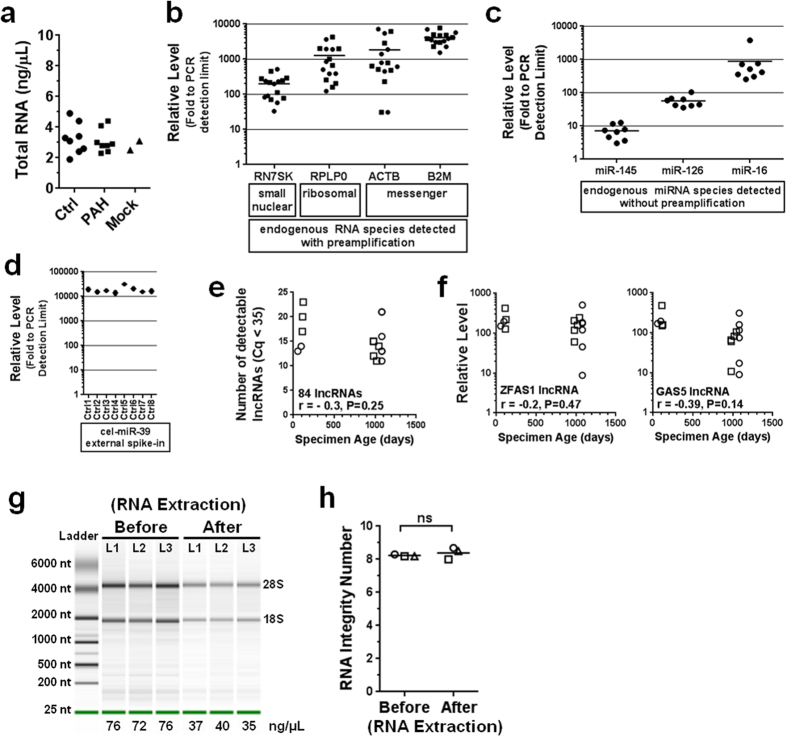
Characterization and confirmation of RNA quality. (**a**) Concentration of total RNA extracted from plasma as measured by Nanodrop spectrophotometry. Two mock RNA extractions performed with ddH2O instead of plasma, highlight the lower limit of detection. (**b**) Relative levels of other endogenous plasma RNA species that were extracted and measured in parallel using the same pre-amplification and RT-PCR platform as the lncRNAs. (**c**) Relative levels of several representative miRNAs that were detected without pre-amplification from the same extracted total RNA. (**d**) A fixed quantity of synthetic 22 nt miRNA mimic (cel-miR-39) with no homology to mammalian miRNAs was spiked into plasma samples just after chemical denaturation of the endogenous RNases. Relative levels are shown for duplicate measurements in eight control subjects. (**e**) No significant correlation observed between the age of plasma specimens and the number of detected lncRNAs. Pearson correlation coefficient and p-value are shown. (**f**) No significant correlation observed between plasma specimen age and two specific lncRNAs that were detectable in all subjects. (**g**) Agilent bioanalzyer electropherogram showing fragment patterns of RNA derived from three different lung tissue specimens (L1-L3) before and after mock RNA extractions. 18s and 28s ribosomal bands are indicated. (**h**) Agilent bioanalyzer RNA integrity numbers are shown for each RNA sample (L1-L3) before and after the mock extractions. Integrity levels vary from 0 (highly degraded) to 10 (highly intact). Circles denote control subjects and squares denote PAH patients.

The observed concentration of total RNA isolated from the plasma samples ranged from 2–5 ng/μL (as determined by Nanodrop, Fig. 2a), which is too low for reliable assessments of RNA integrity through standard techniques such as the electrophoretic quantification of RNA fragment patterns by Agilent Bioanalyzer. However, several lines of empirical evidence argue against RNA degradation as a possible reason for the low lncRNA levels. First, a number of other types of RNAs varying in size and function were robustly detected in the plasma samples at levels between 10–10,000 fold above the assay detection limit (measured in parallel using the same pre-amplification and RT-PCR platform as the lncRNAs), including small nuclear RNA (RN7SK), ribosomal RNA (RPLP0) and messenger RNA (ACTB, B2M) species (Fig. 2b). A selection of 3 common miRNAs known to be enriched in endothelial cells (miR-126) and smooth muscle cells (miR-145), or abundant in plasma (miR-16), were also readily detected without pre-amplification from the same extracted total RNA (Fig. 2c). Second, an external spike-in control RNA was used as an orthogonal approach to assess RNA recovery from plasma, independent of the integrity of the endogenous RNA population. Toward this end, a fixed quantity of a synthetic 22 nt miRNA mimic (cel-miR-39) was spiked into plasma samples just after chemical denaturation of the endogenous RNases. Cel-miR-39 was robustly and consistently detected across these samples (Fig. 2d), providing evidence that even small RNAs (that are generally more difficult to recover than larger RNAs) can be reproducibly recovered using our RNA purification strategy. Third, we observed no significant correlation between the number of detectable lncRNAs and the age of the plasma specimens (Fig. 2e). Similarly, no significant correlation was observed between the levels of two specific lncRNAs that were consistently detectable (ZFAS1 and GAS5), and the age of the plasma samples. Together, these results argue against the possibility that substantial age-dependent RNA degradation may have occurred during storage prior to analysis. Finally, we conducted three mock RNA extractions using known concentrations of total RNA (extracted from human lung tissue) high enough to directly assess RNA integrity before and after the extraction via the Agilent Bioanalyzer. No significant change in the integrity of RNA, as reflected by the Bioanalyzer RNA integrity number (RIN), was observed between samples before (RIN# 8.2 ± 0.1) and after (RIN# 8.4  ±  0.4) the extraction procedure (Fig. 2g,h), and 50 ± 5% of the RNA was recovered after extraction. Collectively, these results indicate that plasma RNAs were not significantly degraded before or during RNA extraction, nor significantly lost during the procedure.

### Internal RT and PCR positive controls confirm no reaction inhibition

**Figure 3:**
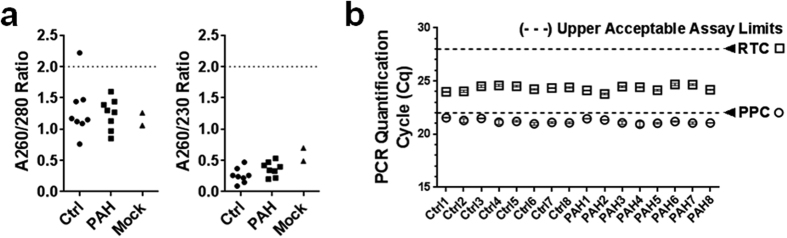
Internal reverse transcription and PCR positive controls confirm no appreciable reaction inhibition, despite low absorbance ratios. (**a**) At low RNA concentrations, typical UV absorbance ratios are not representative of RNA purity. For comparison, 2 mock RNA extractions performed with ddH2O instead of plasma are shown. (**b**) The reverse transcription control (RTC) and positive PCR control (PPC) are fixed quantities of synthetic RNA (with no homology to eukaryotic sequences) and DNA molecules that were integrated into the reverse transcription and PCR reactions of each subject, respectively, and quantified in parallel with the lncRNAs. Cq values for the RTC and PPC of each subject were below manufacturer guidelines (dotted lines), indicative of efficient reverse transcription and PCR reactions.

We next sought to evaluate the purity of the extracted RNA, since contamination with plasma proteins or organic reagents (e.g., phenol, quanidinium isothiocyanate and ethanol) used in the RNA extraction procedure could potentially inhibit subsequent reverse transcription and PCR reactions. At very low RNA concentrations, the standard metrics for assessing RNA purity via UV absorbance ratios (A260/280 and A260/230) may not accurately reflect the true quality and performance of the RNA in downstream enzymatic reactions. In our study, the A260/280 and A260/230 ratios varied from 0.76–2.2 and 0.09–0.53, respectively, which is below typical values of ~2.0 for pure RNA (Fig. 3a). However, the eluate from mock RNA extractions conducted with ddH2O (instead of plasma) yielded a pseudo RNA concentration of 2.5–3 ng/μL and similar purity ratios, illustrating the limitations of UV absorbance measurements under these circumstances (Figs 2a and 3a). To circumvent this issue, internal positive controls were conducted to assess the quality of the reverse transcription and PCR reactions. The reverse transcription control (RTC) and positive PCR control (PPC) are fixed quantities of synthetic RNA (with no homology to eukaryotic sequences) and DNA molecules that were integrated into the reverse transcription and PCR reactions of each subject, respectively, and quantified in parallel with the lncRNAs. Figure 3b shows that the Cq values for both the RTC and PPC of each subject were below manufacturer guidelines, indicative of efficient reverse transcription and PCR reactions. These results suggest that the extracted RNA was free of any significant levels of impurities that might otherwise interfere with the quantification of lncRNAs by RT-PCR. The RTC and PPC control levels were also highly consistent between subjects (i.e., 1% coefficient of variation), indicating that the purity of extracted RNA was highly reproducible.

### Majority of assessed lncRNAs are robustly expressed in pulmonary tissue

**Figure 4:**
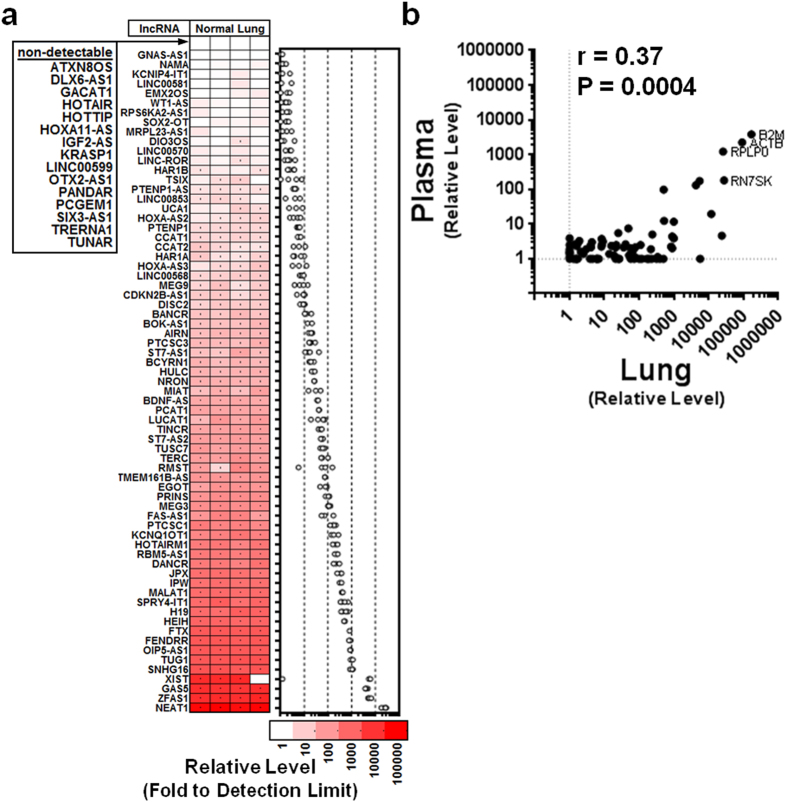
Majority of lncRNAs are robustly expressed in human lung tissue. (**a**) Heatmap showing color coded relative expression levels of the 84 lncRNAs in normal lung tissue specimens from 4 human donors (without preamplification). Each column represents a unique subject and each row represents a different lncRNA, which have been arranged arbitrarily in ascending (mean) abundance. (**b**) The levels of lncRNA and other small RNA species in lung tissue show a small but statistically significant correlation with their mean plasma levels (n = 4 lung samples; n = 8 plasma samples). Spearman correlation coefficient is shown.

The preceding experiments indicate that the low lncRNA levels observed in our human plasma samples were not attributable to technical artifacts. We therefore sought to determine whether there may be inherent biological factors contributing to the low plasma lncRNA levels in healthy and pulmonary hypertension subjects. Because lncRNAs have been reported to be highly tissue specific, we first sought to verify the pulmonary expression of the 84 predefined lncRNA candidates. For this purpose, adjacent normal lung tissue specimens (as identified by routine gross pathological examination) were collected from lung cancer patients undergoing surgical lobectomies. The extracted lung RNA was confirmed to be pure (A260/280 and A260/230 ratios between 2.0–2.16; acceptable RTC and PPC levels) and largely free of degradation (Bioanalyzer RIN#; 7.9–8.5). We found that the majority of lncRNAs (i.e., 76–81%) were detectable without preamplification across four different lung tissue donors. Of the 84 measured lncRNAs, 62 were consistently detected across all subjects, 7 were sporadically detected, and 15 were not detected in any subjects (Fig. 4a). While not conclusive, these results do affirm the relevance of these lncRNAs as potential candidates of dysregulation in PAH, which is a disease characterized by significant pulmonary vascular remodelling. Of note, the detection of these lncRNAs in lung tissue, using the same methods that were used to detect lncRNAs in plasma, provides another positive control that argues against technical artifacts in the previous assessment of circulating lncRNA levels. LncRNA levels varied by ~5 orders of magnitude in the lung tissue specimens, and showed a small but statistically significant correlation with mean plasma lncRNA levels (Spearman r = 0.25, P = 0.02, Fig. 4b). The strength of this correlation increased when other types of RNA species were included in the analysis (RN7SK, RPLP0, ACTB, and B2M; r = 0.37, P = 0.0004).

### Low circulating lncRNA levels are generalizable to other chronic and acute vascular conditions

**Figure 5:**
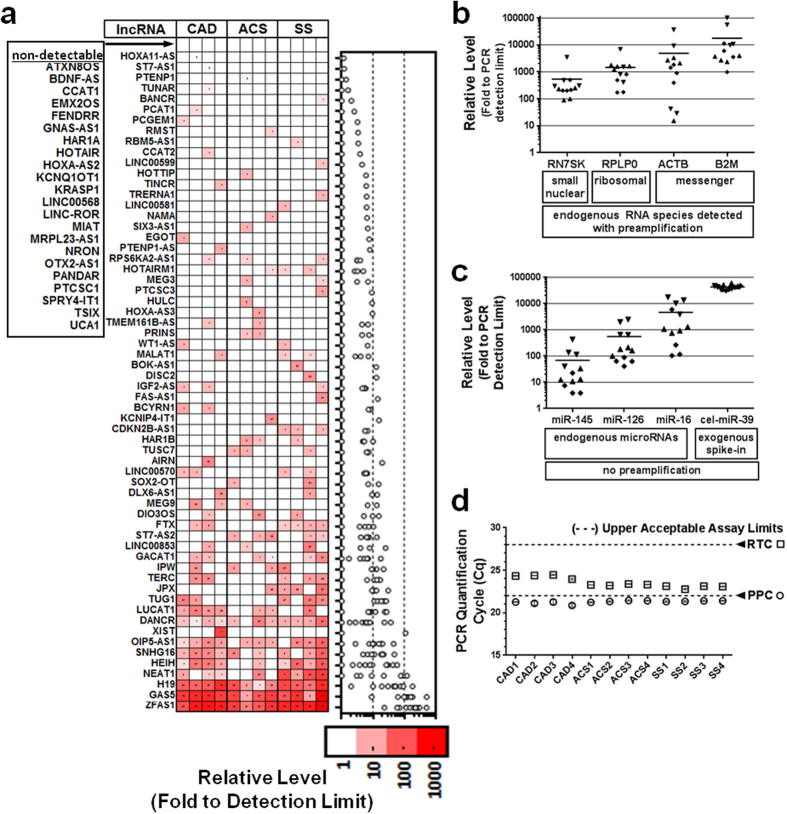
Low and sporadic plasma lncRNA levels are generalizable to other chronic and acute vascular conditions. (**a**) Heatmap showing of relative expression levels of the 84 lncRNAs in patients with coronary artery disease (CAD), acute coronary syndrome (ACS) or septic shock (SS) (n = 4 patients/group). Color coded relative lncRNA levels are expressed fold to the PCR detection limit after log transformation. Each column represents a unique subject and each row represents a different lncRNA, which have been arranged arbitrarily in ascending (mean) abundance. (**b**) Relative levels of other endogenous plasma RNA species that were extracted and measured in parallel using the same pre-amplification and RT-PCR platform as the lncRNAs. (**c**) Relative levels of several representative endogenous miRNAs and an exogenous spiked-in miRNA mimic (cel-miR-39) that were detected without pre-amplification from the same extracted total RNA. (**d**) Internal reverse transcription (RTC) and positive PCR (PPC) controls were quantified in parallel with the lncRNAs. Cq values for the RTC and PPC of each subject were below manufacturer guidelines (dotted lines), indicative of efficient reverse transcription and PCR reactions.

We next sought to determine whether the low circulating lncRNA levels were also generalizable to other clinical conditions. We therefore examined a fairly diverse selection of patients with either coronary artery disease (CAD; n = 4), acute coronary syndrome (ACS; n = 4) or septic shock (SS; n = 4) (Table 1). In contrast to the chronic and more lung specific nature of PAH, other conditions such as CAD and ACS provide insight into both chronic and acute cardiovascular dysfunction, and septic shock is associated with profound physiologic derangements in most organ systems. Of note, the ACS and SS plasma samples were collected independently at two different institutions. Nevertheless, the lncRNA levels in CAD, ACS, and SS patients were generally similar to the PAH and healthy control subjects. Only GAS5, ZFAS1 and H19 lncRNAs were consistently detected across all subjects in these three additional patient cohorts (between 2–515 fold above the PCR detection limit). A total of 59 lncRNAs were detected sporadically at levels less than 100 fold above the PCR detection limit, and 22 lncRNAs were not detected in any of these subjects (Fig. 5a). Of note, the quality and technical performance of the extracted RNA was again validated by the detection of several other types of endogenous circulating RNA species (Fig. 5b,c), exogenous spike-in RNA control (Fig. 5c), and the RTC and PPC positive reaction controls (Fig. 5e). Collectively, these results suggest that the low circulating lncRNA levels are not a disease-specific phenomenon.

### Two of 3 previously reported lncRNA biomarker candidates were not detectable in circulation

**Figure 6:**
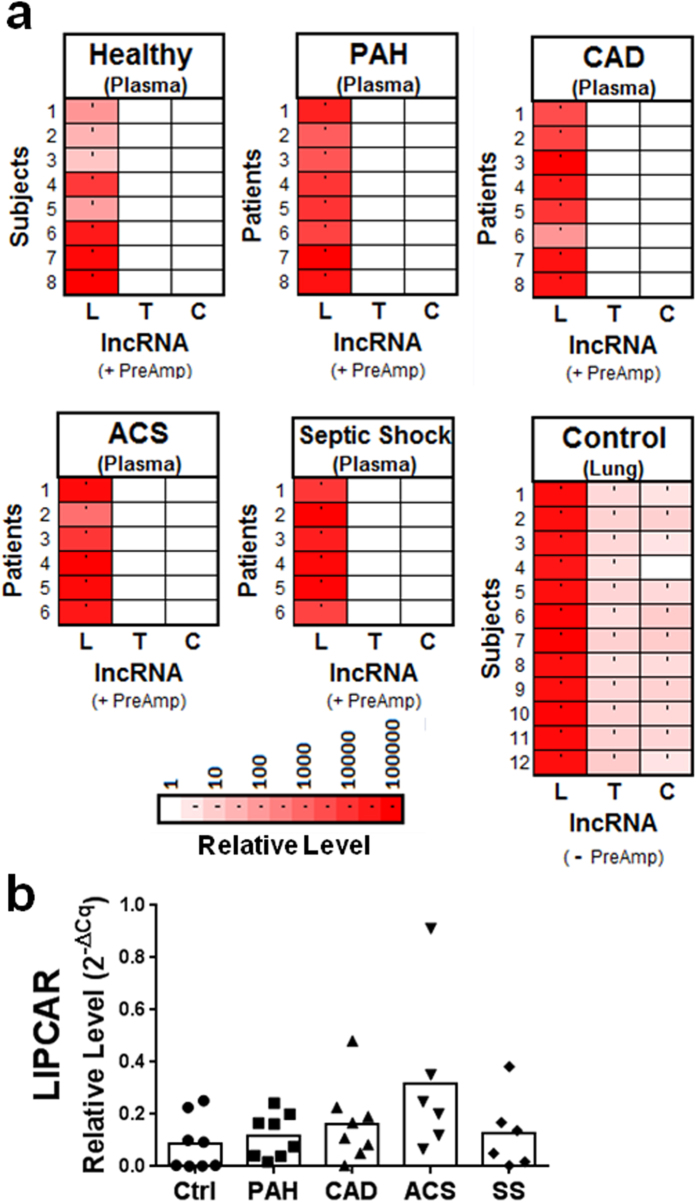
Assessment of previously reported plasma lncRNA biomarkers. (**a**) Heatmaps showing relative levels of Lipcar (L), Tapsaki (T) and Coromarker (C) in plasma from five different groups of subjects or patients, and normal lung tissue specimens. Color coded relative lncRNA levels are expressed fold to the PCR detection limit after log transformation. Each row represents a different subject or patient. Plasma lncRNA levels were quantified after pre-amplification (+PreAmp), while lung levels were quantified without the need for pre-amplification (−PreAmp). (**b**) No significant differences in plasma Lipcar levels observed between groups (Kruskal-Wallis non-parametric test and Dunn’s multiple comparison test). Lipcar levels were normalized to B2M.

We next sought to determine whether the low circulating lncRNA levels were generalizable to other lncRNAs that were not evaluated in our initial screening experiment. For this purpose, we selected three lncRNAs (Lipcar, Tapsaki and Coromarker) that were reported to be elevated in plasma from patients with relevant conditions including heart failure^12^, acute kidney injury^13^ or CAD^14^, respectively, and which have also been proposed as potential biomarkers. Of note, these lncRNAs have no known biological functions, and therefore were not examined in our original screening experiment. Interestingly, TapSaki and Coromarker were not detectable (despite pre-amplification) in any plasma samples across all 5 evaluated subject groups (i.e., healthy, PAH, CAD, ACS or septic shock; n = 6–8 subjects/group; Fig. 6a). Lipcar was detectable in each plasma sample, and ranged from 10 to ~5000 fold above the PCR detection limit (with pre-amplification). While no significant differences in plasma Lipcar levels were observed between groups (Fig. 6b), there appeared to be a nonsignificant increase in ACS patients that might have become significant in a larger sample population. In contrast to plasma levels, all three lncRNAs were detectable in normal human lung tissue specimens, with ~10,000 fold higher levels of Lipcar than TapSaki or Coromarker (Fig. 6a).

## Discussion

This is the first study to explore circulating lncRNAs in PAH patients. We specifically investigated whether 84 lncRNAs representing a strategic selection of candidates that are relatively abundant and functionally well-characterized in cells, were detectable in plasma and altered in PAH patients. No significant differences in plasma lncRNA levels were observed between the PAH and control groups. Furthermore, our results showed that the majority of these lncRNAs were scarcely detectable, despite preamplification prior to qPCR. We demonstrated that these unexpectedly low circulating levels were not an artifact of the quality of the plasma, extracted RNA, or general methodology used in this study, nor specific to only PAH patients. The limited detection of these circulating lncRNAs was also apparent in three additional and quite different clinical conditions, providing evidence of the generality of these observations.

The very limited and sporadic detection of circulating lncRNAs was a surprising finding in this study, given the increasing number of studies that have suggested they might serve as useful biomarkers. We therefore systematically validated the quality of our samples and methods to exclude the possibility that these low levels were due to technical artifacts. First, we demonstrated that several different types of endogenous RNAs (i.e., miRNA, snoRNA, ribosomal RNA and messenger RNA) as well as an exogenous spike-in RNA control (cel-miR-39) were robustly detectable, indicating that the extracted total RNA was not degraded. Second, a post-hoc analysis of lncRNA levels showed no correlation with specimen age, which argues against the likelihood of some partial age-dependent degradation. Third, we conducted mock RNA extractions that demonstrated no decline in total RNA integrity during the extraction procedure, and only a modest loss in the starting RNA mass quantity. This loss would generally be considered negligible because of the exponential amplification afforded by PCR. Finally, integrated positive controls for the downstream reverse transcription and PCR reactions revealed no evidence of reaction inhibition.

To better understand potential biological determinants impacting circulating lncRNA levels, we conducted several further experiments. The pulmonary expression of the 84 lncRNAs was evaluated in human lung tissue, and confirmed that the majority of these lncRNAs were robustly detectable (without pre-amplification) across four different lung tissue donors that were examined. This is an important consideration given that lncRNAs exhibit higher tissue-specificity than protein-coding RNAs^22^. While not definitive evidence, the results do establish the relevance of these lncRNAs as potential candidates for dysregulation in diseases that affect pulmonary structure and function. It is also noteworthy that previous studies have revealed positive correlations between the circulating and lung levels of microRNAs in animal models of PH^23^, suggesting that circulating levels of some RNA species may be driven at least in part by the magnitude of their pulmonary tissue expression. This would be consistent with the fact that the pulmonary vascular bed offers the largest area to release or clear RNAs across the pulmonary circulation, as well as the small but statistically significant correlation between circulating and lung tissue lncRNA levels observed in the current study. In a second experiment aimed at understanding the potential biological factors underlying the low circulating lncRNA levels, we evaluated lncRNA levels in plasma from three further independent sets of patients with either CAD, ACS or septic shock. Again, we found that circulating lncRNA levels were poorly detectable, providing evidence of the generalizability of these observations to both chronic and acute clinical conditions that affect multiple types of organ systems. Finally, we demonstrated that these observations were not limited to only the 84 lncRNAs that were screened in this study. Surprisingly, 2 of 3 previously reported lncRNA biomarker candidates were not detectable in plasma from any of the 5 groups of subjects that were examined in this study, despite pre-amplification measures and stringent quality control checks. This raises questions about the general reproducibility of lncRNA-based biomarkers, and highlights the need for further studies aimed at understanding both the technical and biological determinants that impact circulating lncRNA levels.

Previous studies have reported elevated plasma levels of the lncRNAs Lipcar, TapSaki and Coromarker, in patients with heart failure^12^, acute kidney injury^13^ and coronary artery disease^14^, respectively. In this study we were able to detect lipcar with pre-amplification; however, the other two lncRNAs were undetectable in plasma from healthy subjects or even relevant types of patients with septic shock or CAD. It is known that hemolysis and cellular/platelet contamination of plasma can lead to artificially elevated measurements of microRNAs^24^,^25^, and there is an analogous concern for studies of lncRNAs, given that they are known to be present in blood cells^26^. In addition, the recovery of circulating RNAs, some of which are known to be encapsulated within micro- or nano- vesicles, can be affected by the speed and duration of centrifugation used to prepare the plasma^27^. Therefore, the difference between our findings and prior studies might be due to the preparation and quality of plasma. In this study, we conducted a progressive triple centrifugation strategy to maximize the removal of cells, platelets and cell debris, which might otherwise confound the measurement of lncRNAs derived from cells involved in disease activity (e.g., endothelial, smooth muscle, cardiomyocytes etc). The preparation of plasma specimens used in several prior studies have not been clearly reported^12^,^13^,^14^.

Our demonstration that abundant and functionally relevant cellular lncRNAs were only poorly detectable in circulation has several important implications that will help to inform future studies. First, we now know that an unbiased global assessment of all lncRNAs may be a prerequisite to identifying rare biomarker candidates that are not only altered in circulation, but also readily detectable. Another important finding concerns the relative levels of different RNA species in circulation, which has not previously been characterized. Among the various ribosomal, messenger, small and micro- RNA species that were evaluated in this study, we showed that plasma levels were lowest for lncRNAs. These results provide useful insight for the development of clinical biomarker assays, since an ideal candidate should be readily measurable. Very low expression levels are generally associated with higher levels of variability, which may impact the reliability of a circulating lncRNA-based biomarker. Finally, this study provided information on the relative plasma levels of 84 specific lncRNAs, which could serve as a useful guide for future studies aimed at investigating one or more of these lncRNAs in other specific disease settings.

This study has several potential limitations. One limitation is that we screened only a small fraction of the total number of known lncRNAs, and thus may have overlooked potentially rare biomarker candidates. Our experimental approach, however, was specifically designed to examine lncRNAs with empirically validated functions that might also be leveraged to provide insight into disease mechanisms. This was an important consideration given that the majority of lncRNAs have not been functionally characterized, and could merely represent non-specific transcriptional noise^7^ that is neither functional nor capable of serving as biomarkers. Furthermore, the alternative global microarray screening strategy is not without practical tradeoffs, such as significantly higher costs and sample input requirements that may be difficult with rare and/or limited specimens. Another potential limitation is that we have not attempted to further optimize the lncRNA detection method beyond the specialized preamplification and RT-qPCR kits described herein. Although we used commercially validated kits that offer a relatively high level of intrinsic product standardization and optimization, further investigation of other methodological variables could be beneficial. For instance, scaling up the starting volume of plasma could help improve the lncRNA detection rate, though this strategy may not always be feasible. The archived plasma samples in this study represented limiting reagents, and 200 μL was the maximum volume that could be used consistently across all subjects.

In summary, our study showed that some of the most abundant and functionally well-characterized cellular lncRNAs were only sporadically detectable in plasma. Although these lncRNAs are involved in biological functions that are relevant to known pathologic mechanisms of PAH, their levels were not altered in plasma limiting their usefulness as potential non-invasive biomarkers. This study also provides novel insight into the relative levels of different circulating RNA species, and highlights the additional challenges that must be considered in the potential development of circulating lncRNA-based biomarkers.

## Methods

### Inclusion/Exclusion criteria for human subjects

Peripheral blood samples from PAH patients, CAD patients and healthy subjects were obtained with informed written consent between year 2011–2015. All methods were conducted in accordance with protocols, guidelines and regulations approved by the Ottawa Hospital Research Ethics Board (#2011470-01H). PAH patients were out-patients with a clinical diagnosis of PAH (either idiopathic or associated), and able to provide free and informed consent. Relevant clinical characteristics are summarized in Table 1. CAD patients were out-patients with a clinical diagnosis of stable CAD. Peripheral blood samples from septic shock patients were obtained with informed written consent in 2009 in accordance with protocols, guidelines and regulations approved by the respective research ethics boards in the multi-center Fluid Resuscitation with 5% Albumin versus Normal Saline in Early Septic Shock (PRECISE) pilot trial^28^. Patients were enrolled from the emergency department or intensive care unit after a median of 90 min (IQR; 38–210 min) from their first hypotensive event. Inclusion/exclusion criteria for these patients are as described previously^28^ and relevant clinical characteristics are summarized in Table 1. Peripheral blood samples from ACS patients were obtained with informed written consent between 2009–2013 in accordance with protocols, guidelines and regulations approved by the respective research ethics boards in the multi-center GENESIS-PRAXY study (GENdEr and Sex determInantS of cardiovascular disease: from bench to beyond – PRemature Acute Coronary SYndrome)^29^. Blood samples were collected at the earliest possible time after admission to the coronary care unit for ACS presentation.

### Plasma Isolation

Peripheral blood from PAH, CAD, and healthy volunteers was first drawn into a 3 ml SST tube, which was subsequently discarded to eliminate blood that contacted tissue during venipuncture. Blood was drawn into Becton-Dickinson (BD) vacutainer (sodium citrate) tubes, and centrifuged at 200 × g for 15 min (4 °C). The upper plasma phase was transferred into fresh microfuge tubes, and centrifuged twice at 11,000 × g for 2 min (4 °C) to remove residual cells/platelets/cell debris, with transfer of the plasma supernatant into fresh microfuge tubes between each spin. Plasma was stored at −80 °C. Peripheral blood from septic shock patients was initially drawn into BD vacutainers (sodium citrate), then transferred into a second vessel supplemented with sterile benzamidine (20 mM final concentration), and centrifuged at 1700 × g for 10 min (4 °C). The upper plasma fraction was carefully removed (to within 0.2 mL of the plasma-cell interface) and transferred into cryo tubes for storage at −80 °C. Peripheral blood from ACS patients was drawn into BD vacutainers (sodium citrate) and centrifuged at 3000 rpm for 10 min at room temperature (~20 °C). The upper plasma fraction was carefully transferred into fresh tubes, centrifuged again at 3000 rpm for 10 min at room temperature, then transferred into fresh tubes for storage at −70 °C. Plasma samples showed no evidence of gross hemolysis as evaluated by visual inspection and Nanodrop absorbance measurement at 414 nm (hemoglobin).

### Lung tissue specimen collection

Adjacent normal lung tissue was procured with informed written consent from lung cancer patients (n = 12; 68 ± 9 yr, 78% female) undergoing surgical lobectomies as standard care, and collected upon resection and evaluated in accordance with protocols approved by the Ottawa Hospital Research Ethics Board (Protocol# 20120559-01H). All methods were conducted in accordance with relevant guidelines and regulations. Normal lung tissue was identified by routine gross pathological examinations, excised and initially placed in cold tissue culture media and transferred to the laboratory. The samples were then rinsed in cold PBS, partially sliced to facilitate penetration of RNAlater (Ambion) stabilization reagent, incubated overnight at 4 °C and then stored at −80 °C. The total time from OR collection to stabilization in RNAlater reagent was less than 1.5 hr.

### Total RNA Extraction/Purification

RNA extractions were performed in parallel on the same day for subjects within planned comparison groups, in order to minimize technical variations. Total RNA (including small RNAs) was extracted from 200 μL of citrate-plasma (or ~25 mg of lung tissue) using the miRNeasy mini kit (Qiagen), which uses sequential organic extraction (5:1 ratio of Qiazol:plasma or 1000 uL Qiazol for 25 mg tissue) and silica spin-column purification of total RNA. Of note, the volume of patient plasma was limiting in some cases, such that 200 μL was the maximum volume that could be used consistently across all subjects. Frozen plasma samples were thawed and centrifuged at 11,000 × g for 5 min (4 °C) as an extra precautionary measure to remove any residual cell/debris contaminants prior to RNA extraction. After Qiazol-mediated denaturation of plasma samples, 2 μL of 5 nM cel-miR-39 (Qiagen) was spiked into each plasma sample. An extra on-column DNase digestion step was included in the RNA extraction of lung tissue samples, as recommended by the manufacturer. The total RNA was eluted in a final volume of 50 μL of RNase-free H_2_O.

### RNA Quality Control Assessment

The concentration of total RNA extracted from plasma was generally close to, or below, the manufacturer’s stated detection limit of the Nanodrop 2000 spectrophotometer and the RNA 6000 Nanochip of the Agilent Bioanalyzer. The low RNA concentration precluded the reliable assessment of RNA purity by A260/A280 and A260/A230 absorbance ratios, and it was also too low for assessment of RNA integrity by Agilent Bioanalyzer “RIN” number. To circumvent these issues we utilized the integrated quality controls in the RT^2^ lncRNA RT-PCR system (Qiagen), including the RNA reverse transcription control (RTC) and positive PCR control (PPC) to evaluate the quality of samples. Total RNA extracted from human lung tissue (~300 ng/uL) was confirmed to be pure (A260/280 and A260/230 ratios between 2.0–2.16; acceptable RTC and PPC levels) with minimal degradation (Bioanalyzer RIN#; 7.9–8.5).

### Reverse transcription & PCR

For assessment of circulating lncRNAs, a fixed volume of total RNA extracted from plasma was input into subsequent cDNA reactions (8 uL/rxn), using the RT^2^ lncRNA PreAmp cDNA synthesis kit according to the manufacturer’s instructions (Qiagen). This includes an initial genomic DNA elimination step, isothermal cDNA conversion, and a 12 cycle preamplification (RT^2^ PreAmp PCR master mix and RT^2^ lncRNA PreAmp primer mix) to improve detection of low copy number lncRNAs. Of note, circulating lncRNAs were not detectable without this preamplification strategy, based on a pilot study conducted on a sample of 4 human plasma specimens. The pre-amplified cDNA was then used to quantify 84 predefined lncRNAs (and RN7SK, RPLP0, ACTB and B2M) using the RT^2^ lncRNA PCR Array Human lncFinder (LAHS-001ZE; Qiagen). For assessment of lung tissue levels of lncRNAs, a fixed mass of 800 ng of total RNA was input into the RT^2^ First strand kit (Qiagen) for cDNA conversion (with no pre-amplification). The resulting cDNA was used to assess 84 lncRNAs in the RT^2^ lncRNA PCR Array Human lncFinder. For assessment of plasma levels of Lipcar, Tapsaki and Coromarker, extracted total RNA was pre-amplified using the RT^2^ lncRNA PreAmp cDNA synthesis kit in combination with the RT^2^ PreAmp PCR master mix and primer sequences sourced from their original reports (and synthesized by Integrated DNA Technologies). The preamplified cDNA was then PCR amplified using the RT^2^ SYBR green PCR master mix according to manufacturer instructions. For assessment of circulating miRNAs, a fixed volume of total RNA extracted from plasma was input into the miScript II RT kit for cDNA conversion, and PCR amplified using the miScript SYBR green PCR kit and related primers (Qiagen).

The manufacturer’s recommended PCR cycling conditions for each type of PCR master mix were followed for 40 cycles on a CFX384 PCR machine (Biorad), with a terminal melt curve. PCR reactions were performed in duplicate. Standard negative controls including “no template” and “no reverse transcriptase” reactions were either negative or occasionally exhibited Cq values in excess of 35 (and below 40). Raw PCR Cq values were preprocessed prior to analysis by converting Cq values > 35 to the predefined detection limit of 35, according to manufacturer recommendations. Relative RNA levels expressed fold to the detection limit were calculated using the formula 2^−ΔCq^, where ΔCq = Cq_(target RNA)_ − 35_(detection limit)._

### Statistics

The unpaired student’s t-test (for parametric data) or Mann-Whitney U-test (for non-parametric data) were used for 2-group comparisons. For 3 or more group comparisons, the Kruskal-wallis test was performed with Dunn’s multiple comparison post hoc test (for non-parametric data). Specific statistical tests and measure of variation are specified in each figure legend. Statistical tests were performed with Graphpad Prism V5.0. All tests were performed 2-sided and a significance level of P < 0.05 was considered statistically significant.

## Additional Information

**How to cite this article**: Schlosser, K. *et al*. Assessment of Circulating LncRNAs Under Physiologic and Pathologic Conditions in Humans Reveals Potential Limitations as Biomarkers. *Sci. Rep.* **6**, 36596; doi: 10.1038/srep36596 (2016).

**Publisher’s note**: Springer Nature remains neutral with regard to jurisdictional claims in published maps and institutional affiliations.

## Figures and Tables

**Table 1:** Cohort Characteristics.

Characteristic	Healthy	PAH	CAD		ACS		SS	
			Array	Total	Array	Total	Array	Total
Sample size, n	8	8	4	8	4	6	4	6
Age*, yr	49 ± 6	52 ± 9	53 ± 3	52 ± 4	49 ± 3	49 ± 3	54 ± 12	52 ± 10
Female, n	4	4	4	4	4	6	2	3
Cause of PAH, n (%)								
Idiopathic		3 (38%)						
Associated		5 (63%)						
WHO FC, n*		2.5 ± 0.9						
Hemodynamic parameters* (n)								
mPAP, mm Hg		54 ± 20						
PVR, dsc		620 ± 83						
PCWP, mm Hg		9 ± 5						
ACS Type, n								
STEMI					1	1		
nonSTEMI					2	4		
Unstable Angina					1	1		
APACHE score*							13 ± 5	18 ± 9
28-day mortality, n							0	1

^*^Mean ± STD.

PAH, Pulmonary Arterial Hypertension. CAD, Coronary Artery Disease. ACS, Acute Coronary Syndrome. SS, Septic Shock. WHO FC, World Health Organization Functional Class. mPAP, mean pulmonary artery pressure. PVR, pulmonary vascular resistance. WP, wedge pressure.
